# Genetic Background in Patients with Cancer Therapy-Induced Cardiomyopathy

**DOI:** 10.3390/jcm14041286

**Published:** 2025-02-15

**Authors:** Luca Fazzini, Nicola Campana, Stefano Cossu, Martino Deidda, Cristina Madaudo, Vincenzo Quagliariello, Nicola Maurea, Daniela Di Lisi, Giuseppina Novo, Concetta Zito, Christian Cadeddu Dessalvi

**Affiliations:** 1 Department of Medical Sciences and Public Health, University of Cagliari, 09124 Cagliari, Italy; luca.fazzini10@gmail.com (L.F.); nicolacampana93@gmail.com (N.C.); stefano.cossu94@gmail.com (S.C.); martino.deidda@tiscali.it (M.D.); 2 Cardiology Unit, Department of Health Promotion, Mother and Child Care, Internal Medicine and Medical Specialties (ProMISE), University Hospital ‘Paolo Giaccone’, University of Palermo, 90133 Palermo, Italydanydilis@hotmail.it (D.D.L.); giuseppina.novo@unipa.it (G.N.); 3 Division of Cardiology, Istituto Nazionale Tumori-IRCCS-Fondazione G. Pascale, 80131 Napoli, Italy; quagliariello.enzo@gmail.com (V.Q.); n.maurea@istitutotumori.na.it (N.M.); 4 Cardiology Unit, Department of Clinical and Experimental Medicine, University Hospital “G. Martino”, University of Messina, 98122 Messina, Italy; concetta.zito@unime.it

**Keywords:** cardiotoxicity, genetic, genetic testing, genetic markers, cancer therapy, anthracyclines, cardio-oncology, precision medicine, cardiovascular risk

## Abstract

Emerging evidence indicates that specific genetic variants are associated with an increased risk of toxicity from anticancer treatments and cancer-related cardiovascular complications. These genetic factors influence drug metabolism, efficacy, and susceptibility to adverse effects. For cancer patients, the genetic background can have two major cardiovascular implications, namely therapy-related cardiotoxicity and cancer-related cardiovascular complications. Baseline risk stratification is essential to identify higher-risk individuals and ensure they receive appropriate preventive and therapeutic interventions and more frequent follow-up. Current guidelines recommend stratification based on cardiovascular risk factors, but these factors alone cannot accurately define individual risk. Genetic background has been shown to enhance risk stratification. Beyond rare genetic variants, recent genome-wide association studies have identified single nucleotide polymorphisms implicated in cancer therapy toxicity. Despite their current limitations, polygenic risk scores are expected to play a significant role in risk stratification. This review aims to summarize the current evidence on the role of the genetic background of patients with cancer treated with potentially cardiotoxic drugs who develop cardiotoxicity, aiming to provide insights to refine risk stratification further and tailor the management of these patients.

## 1. Introduction

Significant advancements in early cancer detection and the development of effective treatments have substantially increased long-term survival rates among cancer patients [1,2]. Trend projections indicate that in less than twenty years, the number of new cancer cases globally could rise by almost 50% [3]. The expansion of innovative cancer therapies has improved patient survival, bringing new challenges and highlighting the need for a cardio-oncology program [4,5].

The cornerstone of a cardio-oncology program is the initial assessment of cardiotoxicity risk [6]. Current guidelines suggest a comprehensive evaluation based on clinical features, cardiovascular (CV) history, cardiometabolic risk factors, prior exposure to anticancer therapies, and biomarker levels [7]. The importance of baseline CV risk assessment is underscored by its role in identifying patients who require more intensive CV monitoring during treatment [7]. The risk of cardiotoxicity varies depending on the class of anticancer therapy administered, and specific risk profiles have been developed [8].

Despite the efforts in risk stratification, the currently available tools do not fully capture the real risk of developing cardiotoxic side effects. The phenomenon of cardiotoxicity is influenced by the interplay of several factors including the cancer, the type of anticancer treatment, the potential CV impacts of cancer itself, and the patient’s overall background, which encompasses genetic predispositions [9]. Recent research has emphasized the influence of rare genetic variants and single nucleotide polymorphisms (SNPs), demonstrating that a patient’s genetic background can modulate the predisposition to cardiotoxicity from anticancer therapies and may also affect cancer-associated CV complications, including thromboembolic events [10,11]. Indeed, the understanding of the genetic background’s impact on cardiotoxicity is still at the beginning and, despite the CV risk assessment tools, the early identification of high-risk individuals is still suboptimal.

This review aims to summarize the current evidence on the genetic factors influencing susceptibility to CV toxicities from cancer therapies, including both xenobiotics and targeted/biological therapies, in this rapidly evolving field. PubMed was the electronic database screened to identify relevant manuscripts using the following search terms: cardiotoxicity, anthracycline-induced cardiomyopathy, chemotherapy-induced cardiomyopathy, cancer therapy-related cardiovascular toxicity, genetic testing, genetic background, genetic variant, single nucleotide polymorphism, and polygenic risk scores. Additionally, the reference lists of the identified articles were reviewed to find further relevant studies. The authors then selected the most pertinent and informative studies for the discussion of the topic.

## 2. Pathogenic Mechanisms of Cancer Therapy-Related Cardiotoxicity

According to the latest European Society of Cardiology Cardio-Oncology guidelines, cancer therapy-related cardiac dysfunction is the recommended umbrella term for cardiotoxicity [7]. This condition can be symptomatic, ranging from mild heart failure (HF) symptoms to the need for inotropic support or mechanical circulatory assistance, or asymptomatic, encompassing significant changes in global longitudinal strain or a decline in left ventricular ejection fraction.

The main chemotherapy drugs responsible for CV damage are anthracyclines, tyrosine-kinase inhibitors (TKIs), immune checkpoint inhibitors (ICIs), fluoropyramides, vascular endothelial growth factor (VEGF) inhibitors, and anti-HER2 drugs. [12,13]. The net cardiotoxic effect results from the interaction among several cardiotoxic pathways, which are influenced by factors including individual susceptibility, sex differences, ethnic differences, metabolic response to cardiotoxic therapy, and administered cumulative dose [14,15,16,17,18,19]. A summary of the main mechanisms of cancer therapy-induced cardiotoxicity is provided below (Table 1).

### 2.1. Oxidative Stress

Oxidative stress results from an imbalance between the production of reactive oxygen species (ROS) and reactive nitrogen species (RNS) and cellular defense mechanisms. ROS and RNS play a significant role in signaling pathways in several cell populations, among others, in cardiomyocytes. Importantly, the myocardium lacks antioxidant agents, leading to a near-maximal saturation of antioxidant defenses in cardiomyocytes at a steady state [20]. Consequently, the myocardium is very vulnerable to oxidative stress-induced damage. The main pathogenic mechanisms of anthracyclines involve the generation of free radicals, the promotion of mitochondrial ROS production by impairing mitochondrial function [21,22], and ROS generation by complexes formed from anthracycline and iron that undergo redox cycling [22].

### 2.2. Topoisomerase 2-β Inhibition

DNA topoisomerase 2 (Top2) has two isoforms, Top2-α and Top2- β, and the latter is the only isoform expressed in adult mammalian cardiomyocytes. Top2 facilitates the transient cleavage and ligation of the DNA double strand during DNA replication and transcription [23]. Especially doxorubicin, among anthracyclines, which binds to Top2-β, inducing DNA and mitochondrial damage and oxidative stress, which are their both anticancer and cardiotoxic mechanisms. Furthermore, inhibiting Top2-β could be a promising primary prevention strategy [24].

### 2.3. Inflammation

Inflammation plays a crucial role in cardiotoxicity caused by anticancer treatments. Specifically, doxorubicin induces cardiac damage by activating some inflammatory pathways in cardiomyocytes, leading to an increase in cytokines IL-1 and TNF-α, and p38-MAPK and NF-κB activation [13]. Moreover, Toll-like receptor TLR4 and TLR2 expression is higher in patients under doxorubicin therapy, which may suggest a predisposition to myocardial damage and a higher sensitivity to doxorubicin’s cardiac effects [25].

### 2.4. Endothelial Dysfunction

Endothelial dysfunction frequently leads to vascular toxicity, a common adverse effect of current anticancer drugs. VEGF inhibitors, anthracyclines, plant alkaloids, alkylating agents, and radiotherapy potentially cause vascular damage [26]. Chemotherapy drugs interfere with endothelium signaling pathways by altering gene expression, reducing nitric oxide (NO) bioavailability, and promoting ROS generation. Additionally, they induce the production of damage-associated molecular patterns (DAMPs) responsible for both endothelial and leukocyte activation, resulting in systemic and local inflammation, cytokine increase, and leukocyte diapedesis [27].

### 2.5. Apoptosis and Autophagy

Several anticancer drugs can promote cellular death pathways, such as caspase and apoptosis pathways [28]. Recently, it has been shown that some chemotherapy drugs, such as anthracycline, could cause dysregulation in the autophagy mechanism, facilitated in the early phase and suppressed in the late phase of treatment, leading to a decreased elimination of damaged mitochondria and ultimately promoting cardiotoxicity [28].

### 2.6. Mitochondrial Dysfunction

ROS, as anticipated above, may determine mitochondrial dysfunction in cells with high metabolic activity, such as cardiomyocytes. Anthracyclines have a dose-dependent effect on increasing mitochondrial membrane permeability, and such a mechanism is likely to be influenced by calcium, redox reactions, and mitochondrial iron levels. The alteration in mitochondrial membrane permeability also contributes to release of pro-apoptotic factors not related to the caspase pathway, a mechanism known as ferroptosis [29].

## 3. Genetics and Cancer Therapy-Related Cardiotoxicity

### 3.1. Rare Genetic Variants Associated with Cancer Therapy-Induced Cardiomyopathy

Based on minor allele frequency (MAF), genetic variants can be summarized into two classes, namely common (typically MAF > 5%) and rare (MAF < 0.1%) variants [9]. Both rare and common genetic variants are likely to impact the baseline risk of developing cardiotoxicity, which is currently captured by clinical risk factor assessment, including demographics, a history of CV disease, cardiometabolic risk factors, prior anticancer therapy, a history of cardiotoxicity, and biomarkers [7,10]. Piecing together risk factors with the individual genetic background is likely to increase the yield of cardiotoxicity development prediction in patients who receive chemotherapy.

Several common genetic variants have been identified to be implied to either increase or reduce the risk of cancer therapy-induced cardiomyopathy (CCM) and are discussed below. Rare monogenic variants were recently explored in patients’ cohorts affected by CCM. Specifically, the *TTN* gene encodes the titin, the most important sarcomere scaffold and regulatory factor for cardiac contraction. *TTN* truncating variants (*TTN*tvs) are the most common rare genetic variants associated with dilated cardiomyopathy (DCM), with detection rates of 25% in familial cases, being slightly lower in sporadic cases [30]. In addition, these mutations are present in a significant proportion of peripartum and alcoholic cardiomyopathy [31,32], suggesting that some cardiomyopathies could share a genetic background. Thus, people with *TTN*tv mutation are more susceptible to HF during trigger situations, such as pregnancy or cancer therapy. Garcia-Pavia et al. included 213 patients with CCM from three cohorts as follows: 99 adults with diverse cancers (retrospective), 73 phenotyped adults with breast cancer (prospective), and 41 phenotyped children with acute myeloid leukemia (prospective) [33]. Cardiomyopathy genes, including nine prespecified DCM genes including BCL2-associated athanogene-3 (*BAG3*), desmoplakin (*DSP*), *LMNA*, *MYH7*, sodium voltage-gated channel, alpha subunit 5 (*SCN5A*), telethonin (*TCAP*), cardiac troponin C (*TNNC1*), *TNNT2*, and *TTN*, were sequenced. Among nine prioritized genes, patients with CCM had more rare protein-altering variants than comparative cohorts, and it was found that *TTN*tvs predominated, occurring in 7.5% of patients with CCM compared to 0.6% of the reference population. Importantly, adult patients with CCM and *TTN*tvs had a higher risk of cardiac dysfunction and supraventricular arrhythmias, including atrial fibrillation [33].

Recently, a case–control study retrospectively included 46 patients with anthracycline-induced cardiomyopathy. The diagnostic yield of genetic testing was evaluated in comparison to a group of 46 patients with DCM and a matched cohort of 111 individuals without heart disease. A total of 59 genes were tested. A total of 63% of CCM patients were found to be genetic variant carriers (pathogenic, likely pathogenic, or variants of uncertain significance); while a similar proportion was found in the DCM cohort (73.9%), a significantly higher proportion compared to the control cohort (39.6%) was observed. *TTN*tvs turned out to be the most frequent mutation in CCM patients (43% of all variants) [34]. These main studies on rare genetic variants are briefly summarized in Table 2.

*TTN*tvs have been demonstrated to be an emerging theme for genetic predisposition to CCM. Thus, *TTN*tv carriers may be at higher risk for developing CCM. These observations could possibly indicate that a genetically vulnerable but otherwise healthy myocardium may be more susceptible to damage if exposed to cancer therapy drugs.

### 3.2. Single Nucleotide Polymorphisms and Polygenic Risk Scores

CV diseases have a diverse genetic basis, ranging from familial monogenic causes of HF with variable penetrance to genetic variants with lower penetrance that interact with multiple environmental factors. Notably, family members may develop the disease even in the absence of monogenic variants, highlighting the largely polygenic nature of CV disease development [35,36]. This polygenic foundation can be assessed by aggregating the weighted effects of disease-associated SNPs across the entire genome, identified through genome-wide association studies (GWASs) into what is known as a polygenic risk score (PRS). The PRS provides a quantitative risk assessment by accounting for common genetic variants with small effect sizes [37,38]. The relevance of SNPs and PRSs has been explored in the context of HF [39,40,41], including their role in susceptibility to cardiotoxicity.

#### 3.2.1. Single Nucleotide Polymorphisms in Drug Transporters and Metabolizer-Involved Genes

Anthracyclines and other anticancer therapies require active transport by proteins that serve as the cellular transporters of multiple drugs. Several genes encoding drug transporters and drug metabolizers have been identified as involved in cardiotoxicity, including adenosine triphosphate-binding cassette transporter (*ABC*) and carbonyl reductase 3 (*CRB3*), respectively [9]. *ABC* proteins are among the major families involved in drug transport, and over the past twenty years, several polymorphisms in this gene family have been correlated with a higher likelihood of developing cardiac toxicity in both pediatric and adult patients [9,42,43,44], while other SNPs appear to be protective by enhancing drug elimination in cardiomyocytes [45]. Similarly, Visscher et al. identified several SNPs in a gene family of solute carriers (SLCs) that play a protective role against anthracycline-induced cardiotoxicity [44,46]. Regarding drug metabolism, anthracyclines undergo multiple metabolic pathways, including cytochrome P450 reduction, NADH dehydrogenase, and nitric oxide synthase, leading to their conversion into intermediate molecules and reactive radicals. Genetic variants in the enzymes involved in these processes can influence drug metabolism. Variants in the enzymes involved in such processes may alter drug metabolism. In particular, the CRB3 gene encodes an enzyme that converts doxorubicin into doxorubicinol, a cardiotoxic metabolite [47]. SNPs in the *CRB3* gene have been associated with cardiotoxicity in both pediatric survivors and adults with breast cancer receiving low-dose doxorubicin [45,48].

#### 3.2.2. Single Nucleotide Polymorphisms in Oxidative Stress Response-Involved Genes

Beyond drug transport and metabolism, SNPs associated with cardiotoxicity have been identified in genes encoding proteins involved in oxidative stress response (e.g., *HAS3* and *GSTM1*, which encode hyaluronan synthase 3 and glutathione S-transferase Mu-1, respectively) [49,50], in iron metabolism (*HFE* gene, which regulates iron homeostasis) [51], and in topoisomerase-mediated DNA damage (*RARG* gene, which encodes for retinoic acid receptor gamma) [52,53].

#### 3.2.3. GWAS and PRSs

In a GWAS conducted by Serie et al., cancer patients treated with adjuvant doxorubicin and cyclophosphamide, followed by randomization to either weekly paclitaxel or paclitaxel plus trastuzumab, showed that SNPs in *LDB2*, *BRINP1*, chr6 intergenic, *RAB22A*, *TRPC6*, and *LINC01060* genes were associated with cardiotoxicity, as assessed by the decline in left ventricular ejection fraction during echocardiography in the trastuzumab-treated arm [54]. These genes encode proteins involved in various pathways, including cytoskeletal organization (*LDB2*, *TRPC6*), calcium signaling (*TRPC6*), vesicle trafficking (*RAB22A*), and neural differentiation (*BRINP1*), while *LINC01060* is a long non-coding RNA implicated in transcriptional regulation. Numerous other SNPs have been correlated with cardiotoxicity in both trastuzumab- [55,56,57,58] and anthracycline-treated cancer patients [59,60,61,62] involving complex receptor–ligand signaling abnormalities and intricate molecular pathways.

Over the years, GWASs have identified SNPs in genes implicated in oxidative stress, drug transport, drug metabolism, mitochondrial dysfunction, and DNA damage response. However, the effect sizes of individual SNPs are generally weak, limiting their clinical applicability. Consequently, PRSs, which aggregate the weighted effects of multiple SNPs, provide a more comprehensive quantitative risk assessment. Sapkota et al. assessed the role of a PRS in estimating the risk of developing coronary artery disease among childhood cancer survivors. The proposed PRS was able to identify individuals at higher risk who may benefit from targeted screening [63]. Nevertheless, PRSs specifically designed for predicting cardiotoxicity from anticancer therapies are still lacking. The *P2RX7* gene encodes the receptor *P2X7*, a ligand-gated ion channel involved in adenosine triphosphate (ATP)-mediated signaling, playing a role in inflammation, apoptosis, and immune responses. Sharafeldin et al. combined multiple SNPs identified in the *P2RX7* gene, showing that together they exert a much larger quantitative effect compared to individual SNPs [64]. Although this does not constitute a validated PRS, combining individual SNPs appears to be a promising approach for providing risk assessments applicable at the individual patient level. Recently, an Australian research group compared a clinical prediction HF model to a previously validated HF prediction PRS (PRS-HF) in a cancer population [65]. The PRS-HF underperformed the clinical score in predicting HF among cancer survivors. While combining the clinical score with the PRS-HF did not enhance prediction performance, including the PRS-HF in a multivariable analysis with other HF risk factors significantly improved predictive accuracy compared to a model including only the clinical score and the PRS-HF [65]. In summary, this study describes the clinical score outperforming the PRS-HF in predicting HF, highlighting the current limitations of PRSs and raising awareness for the need of future research to design PRSs capable of predicting cardiac toxicity in cancer survivors.

### 3.3. Anthracycline-Related Toxicity and Genetics

We previously discussed the current evidence on both rare genetic variants and SNPs in anticancer therapy cardiotoxicity, including anthracyclines. Given that anthracycline represents one of the most common anticancer therapies administered in adults and children, we aim to further summarize the current evidence of the genetics’ impact on anthracycline-induced cardiotoxicity. It is likely that some individuals carry an innate genetic predisposition to present cardiac toxicity, shedding light on the reasons why some individuals develop cardiac dysfunction while others do not. A recently published case report described a 6-year-old girl with high-grade osteosarcoma treated with doxorubicin who developed severe left ventricular dysfunction with HF. Considering the clinical course severity and acuity, she received only a fraction of her anthracycline dose, and a genetic background was hypothesized. She underwent whole exome sequencing (WES), which identified three variants related to anthracycline-induced cardiomyopathy that likely influenced the clinical and cellular response in the young girl [66]. Although a single case report cannot explore and reveal causality, this supports the relationship between genetics and cardiotoxicity. A larger study including 280 patients treated for childhood cancer performed a GWAS seeking variants associated with anthracycline-induced cardiotoxicity defined by both asymptomatic cardiac dysfunction and HF. A variant in the *RARG* gene (rs2229774, p.Ser427Leu) was identified to be highly associated with anthracycline-induced cardiotoxicity. More recently, haptoglobin-specific alleles showed a correlation to anthracycline-induced cardiotoxicity development in childhood cancer survivors [67]. Both rare genetic variants and SNPs appear to play a role in determining anthracycline-induced cardiotoxicity. Given the widespread use of anthracyclines across both pediatric and adult populations, coupled with the increasing incidence of cancer diagnoses, ongoing research into these genetic factors is essential for identifying patients at heightened risk.

## 4. Role and Timing of Genetic Testing: Who Should Receive Genetic Testing?

Given the limited evidence to date, the European Society of Cardiology’s current guideline on cardio-oncology does not recommend routine genetic testing for cardiotoxicity risk stratification in patients with malignancy before the initiation of therapies. A personalized genetic approach is hypothesized to support individual susceptibility to CV diseases in patients with cancer in the future. Additionally, it is reasonable to expect that adult and childhood cancer survivors might be distinguished in risk assessments given the significantly higher risk of CV mortality observed in childhood and adolescent cancer survivors as they reach young adulthood [68].

## 5. Genetics and Cancer-Related Cardiovascular Complications

### 5.1. Thromboembolic Complications (JAK2, K-ras, Factor V Leiden, and Tumors)

It has long been known that several cancer-related factors, such as hypercoagulability, inflammation, and paraneoplastic syndrome, are believed to play a significant role in the development of CV diseases. However, it is only recently that dedicated studies have begun to evaluate the correlation between genetic background and susceptibility to cancer-related CV complications.

Several studies have evaluated the predisposition to developing cancer-associated venous thromboembolism (VTE) in relation to the presence of specific mutations across various types of tumors [69] (Table 3). Regarding non-small-cell lung cancer (NSCLC), multiple studies have shown that patients with rearrangements in the anaplastic lymphoma kinase (*ALK*) and c-ros oncogene 1 (*ROS1*) genes are at an increased risk of VTE events [70,71,72,73,74]. In the context of primary brain tumors, an increased rate of VTE has been demonstrated in patients with gliomas that exhibited high expression of podoplanin and the presence of wild-type IDH1/2 (which is often mutated in this type of tumor) [75,76]. Additionally, it is important to note that the risk increased exponentially when both conditions were present [77]. The presence of a *KRAS* mutation in patients with colon cancer significantly increases the risk of VTE [78].

The assessment of cancer-associated thrombosis risk can be evaluated regardless of the tumor type based solely on the specific mutation. Dedicated studies have shown an increased rate of arterial thromboembolism and VTE within one year in patients with *KRAS* and *STK11* mutations, regardless of the tumor site [79,80]. Based on this evidence, the “ONCOTHROMB” score has recently been developed. By analyzing nine genetic variants, tumor site, TNM stage, and the patient’s BMI, it is able to predict the onset of VTE with greater accuracy compared to the Khorana score, which is the main score currently used in clinical practice [81].

### 5.2. Paraneoplastic Syndromes

It has been well-established for years within the scientific community that the development of neoplastic pathology is associated with the occurrence of paraneoplastic syndromes, characterized by symptoms manifesting in areas distant from the tumor or its metastases. These symptoms, which may affect any organ system, can arise either from substances secreted by the tumor or as a result of cross-reactivity, where antibodies directed against tumor antigens also target healthy tissues. Currently, there is limited evidence in the literature regarding the role of genetic background in the development of CV paraneoplastic syndromes, including hypertension, atrial fibrillation, and HF.

Research on genetic susceptibility to hypertension in cancer patients is scarce. A study highlights how the *BRCA* gene mutation linked to breast cancer can also cause endothelial damage and increased blood pressure [82]. Additionally, the GRK4 gene, associated with breast cancer, may contribute to hypertension in healthy individuals. Future research might explore the connection between these genetic mutations and hypertension in breast cancer patients.

When discussing genetic predisposition to CV diseases in cancer patients, it is necessary to discuss the role of clonal hematopoiesis of indeterminate potential (CHIP). CHIP arises from somatic mutations in hematopoietic stem cells, leading to clonal progeny of mutant leukocytes in the blood [83]. These mutations impart a pro-inflammatory effect on immune cells, contributing to a pro-atherogenic environment and promoting the onset of HF [83,84]. Both patients with hematologic malignancies and those with solid tumors have higher rates of CHIP compared to the general population [85,86]. Furthermore, evidence suggests that healthy individuals with CHIP have an increased risk of developing hematologic cancers compared to those without CHIP [85]. The presence of CHIP doubles the risk of coronary heart disease and ischemic stroke [85]. CHIP has also been linked to the development of HF, both of ischemic and non-ischemic origin [87]. The presence of CHIP carries prognostic significance, as patients with CHIP exhibit a higher rate of disease progression, regardless of etiology [88]. Additionally, a study demonstrated that among HF patients, CHIP was more frequent in a cohort with cardiogenic shock compared to a cohort with stable HF [89].

## 6. Future Perspectives

The genetic background of a patient appears to influence the likelihood of developing cardiotoxicity and CV complications related to cancer therapies, either by increasing or reducing their risk. To enhance the clinical applicability of genetic testing, future research should prioritize integrating rare genetic variants and PRSs with existing patient and treatment-related risk factors, aiming to refine individualized HF risk prediction in cancer patients. This approach is essential to evaluate whether genetic testing can offer superior risk stratification compared to models based solely on clinical factors.

### 6.1. Potential Clinical Application

The combination of genetic and clinical data should be optimized using advanced bioinformatics tools and machine learning. Recently, a risk prediction model for late anthracycline-induced cardiotoxicity in childhood cancer survivors was developed, combining clinical and genetic factors [90]. The study identified variants in cardiac injury pathway genes that confer protection against cardiotoxicity. By combining these genetic findings with clinical risk predictors, the researchers developed a prediction model, demonstrating its superiority over the clinical-only model in predicting cardiotoxicity. These technologies could unlock multiple clinical applications for patients at a heightened risk of CCM, (i) enabling closer monitoring with biomarkers and echocardiographic assessments with different screening protocols based on the baseline individualized risk, (ii) allowing for cardiotoxic therapy dose adjustment, and (iii) evaluating the potential use of pre-emptive cardioprotective treatments to mitigate risk.

Beyond managing modifiable CV risk factors, including hypertension, hypercholesterolemia, diabetes mellitus, and smoking cessation, there is also emerging evidence supporting the use of cardioprotective medications typically employed in HF management with controversial results [91]. Specifically, sodium–glucose cotransporter 2 inhibitors have proven effective across the full spectrum of HF ejection fraction. Their recently proposed beneficial effects on endothelial function, oxidative stress, and nitric oxide pathways make this drug class a promising candidate for testing in the context of cardiotoxicity prevention [92].

The use of cardioprotective medications in a preventive context for patients identified as high risk represents an important frontier in research.

### 6.2. Limitations and Challenges

However, several challenges must be addressed, including the high cost of genetic testing without well-established cost-effectiveness benefits, issues of accessibility, and the necessity of involving genetic counselors to navigate ethical considerations and provide accurate counseling and guidance on results interpretation. Additionally, a significant limitation is the difficulty of translating findings from GWASs into clinical practice. While GWASs have identified several genetic variants associated with cardiotoxicity, these findings often lack sufficient reproducibility and clinical applicability. Finally, emerging circulating biomarkers, including microRNAs with a cardiac origin, have shown to correlate with cardiotoxicity [93]. However, their applicability in clinical routine is still limited due to high cost and poor sample analysis standardization. Integrating currently used biomarkers, such as Troponin and natriuretic peptides, emerging biomarkers, and genetic data would likely provide a more comprehensive tailored patient risk assessment. There is still a considerable gap in understanding how these factors can be used to predict individual patient risk accurately and how to integrate these findings with other clinical variables for more effective decision-making. In the future, PRS development and the integration of diverse data sources may partially address this limitation.

In conclusion, genetic risk models integrated with detailed clinical data could inform preventive strategies, ultimately supporting the tailored long-term monitoring of HF risk in cancer patients.

## Figures and Tables

**Table 1:** Summary of cancer therapy-induced cardiotoxicity pathogenetic mechanisms.

Pathogenic Mechanism	Molecular Agents	Ultimate Effects
Oxidative stress	ROS, RNS	Cellular death, gene expression alteration, cellular hypertrophy, impaired mitochondrial function, endothelial damage
Topoisomerase inhibition	Topoisomerase 2-β	Mitochondrial dysfunction, transient braking and adjoining of DNA, changes in transcriptome, ROS production
Inflammation	Cytokines IL-1, TNF-α, ILR4, TLR2	Cardiomyocyte damage, endothelial dysfunction and damage
Endothelial dysfunction	ROS, reduction in NO, DAMPs, cytokines	Vascular inflammation and damage, leukocyte transmigration
Apoptosis and autophagy	Death receptors (DR4, DR5), caspase	Activation of apoptosis and autophagy
Mitochondrial dysfunction	ROS, calcium and mitochondrial iron	Impaired mitochondrial function, cellular death

**Table 2:** Summary of studies that explored the prevalence of rare genetic variants in patients with cancer therapy-induced cardiomyopathy.

Author	Year	Cohort	Findings
Pablo Garcia-Pavia et al. [33]	2019	213 patients with CCM (anthracycline and/or anti-HER2 induced): 99 with diverse cancers, 73 adults with breast cancer, 41 children with acute myeloid leukemia	*TTN*vts the most frequent variant (7.5%), associated with higher risk of heart failure, atrial fibrillation, and impaired myocardial recovery
Hanne M Boen et al. [34]	2024	46 CCM (anthracycline induced), 46 DCM, 111 patients without cardiac disease	*TTN*vts the most frequent variant in CCM cohort (43%), no significant differences in severity of CCM and in recovery rate in variant-harboring individuals versus non-variant-harboring individuals

CCM: cancer therapy-induced cardiomyopathy; DCM: dilated cardiomyopathy; TTN: titin.

**Table 3:** Summary of the studies that explored the role of genetic background in the development of thrombosis among cancer patients.

Title, Author, and Year of Publication	Tumor Type	Gene	Gene Function
Feifei Dou et al., 2019 [70]	Non-small-cell lung cancer	*ALK*	The *ALK* gene encodes the ALK receptor tyrosine kinase. The main molecular pathways activated by the receptor (PI3K/AKT/mTOR, ERK, JAK/STAT, SHC/GRB2) are involved in the regulation of cell proliferation, differentiation, and survival, particularly in neuronal cells. The most common mutations of the gene arise from gene fusions (the most frequent being the fusion between the *ALK* gene and *EML4*), leading to the production of chimeric proteins. This results in the constant activation of cellular molecular pathways, causing uncontrolled proliferation and resistance to apoptosis.
Hanny Al-Samkari et al., 2020 [71]	Non-small-cell lung cancer	*ALK*	
Zugazagoitia J et al., 2018 [72]	Non-small-cell lung cancer	*ALK*	
Chiari et al., 2020 [73]	Non-small-cell lung cancer	*ROS-1*	The *ROS-1* gene codes for the ROS1 protein, a receptor tyrosine kinase. This receptor is involved in cellular signaling, regulating critical processes such as cell growth, proliferation, differentiation, and survival. Gene mutations can lead to *ROS1* fusion with other genes, forming abnormal chimeric proteins that maintain their kinase activity in an uncontrolled manner.
Terry L. Ng et al., 2018 [74]	Non-small-cell lung cancer	*ROS-1*	
Dusten Unruh et al., 2016 [76]	Gliomas	*Isocitrate dehydrogenase 1 (IDH1)*	Isocitrate dehydrogenase 1 (IDH1) is a key enzyme involved in cellular metabolism, particularly in the citric acid cycle. Mutations in the gene result in the accumulation of the metabolite D-2-hydroxyglutarate (D-2-HG). Mutant IDH1 has potent antithrombotic activity.
S. Ades et al., 2014 [78]	Colon rectal cancer	*KRAS*	The *KRAS* gene codes for the KRAS protein, which when bound to GTP, activates intracellular signaling pathways like MAPK/ERK and PI3K/AKT, promoting cell proliferation. Mutations in the gene cause KRAS to remain permanently bound to GTP, leading to uncontrolled cell growth and contributing to cancer development.

## Abbreviations

The following abbreviations are used in this manuscript:

ABC	Adenosine triphosphate-binding cassette transporter
BAG3	BCL2-associated athanogene-3
CCM	Cancer therapy-induced cardiomyopathy
CRB3	Carbonyl reductase 3
CV	Cardiovascular
DCM	Dilated cardiomyopathy
DAMPs	Damage-associated molecular patterns
DR	Death receptor
DSP	Desmoplakin
HAS3	Hyaluronan synthase 3
HF	Heart failure
HFE	Gene regulating iron homeostasis
HER-2	Human epidermal growth factor receptor 2
ICIs	Immune checkpoint inhibitors
IL-1	Interleukin 1
LDB2	LIM domain-binding protein 2
LMNA	Lamin A/C
MAPK	Mitogen-activated protein kinase
MAF	Minor allele frequency
MYH7	Myosin heavy chain 7
NF-κB	Nuclear factor kappa B
NO	Nitric oxide
PRS	Polygenic risk score
ROS	Reactive oxygen species
RNS	Reactive nitrogen species
SCN5A	Sodium voltage-gated channel, alpha subunit 5
SLCs	Solute carriers
SNPs	Single nucleotide polymorphisms
TCAP	Telethonin
TNF-α	Tumor necrosis factor-alpha
TNNC1	Cardiac troponin C
TNNT2	Troponin T2
Top2	Topoisomerase 2
TTN	Titin
TTNtvs	TTN truncating variants
TLR	Toll-like receptor
VEGF	Vascular endothelial growth factor
WES	Whole exome sequencing
